# A Review on Tramiprosate (Homotaurine) in Alzheimer's Disease and Other Neurocognitive Disorders

**DOI:** 10.3389/fneur.2020.00614

**Published:** 2020-07-07

**Authors:** Sagrario Manzano, Luis Agüera, Miquel Aguilar, Javier Olazarán

**Affiliations:** ^1^Department of Neurology, Infanta Leonor Hospital, Madrid, Spain; ^2^Centro de Investigación Biomédica en Red de Salud Mental (CIBERSAM), Instituto de Investigación Hospital 12 de Octubre (i+12), Madrid, Spain; ^3^Unit of Neurodegenerative Diseases DOMUS-Vi, Department of Neurology - Àptima Terrassa, Barcelona, Spain; ^4^Memory Disorders Unit, HM Hospitals & Neurology Service, Gregorio Marañón Hospital, Madrid, Spain

**Keywords:** homotaurine, tramiprosate, amyloid, Alzheimer's disease, neurocognitive disorders, neurodegenerative diseases

## Abstract

Alzheimer's disease (AD) is the most prevalent neurodegenerative condition, especially among elderly people. The presence of cortical β-amyloid deposition, together with tau phosphorylation and intracellular accumulation of neurofibrillary tangles (NFT) is the main neuropathologic criteria for AD diagnosis. Additionally, a role of inflammatory, mitochondrial, and metabolic factors has been suggested. Tramiprosate binds to soluble amyloid, thus inhibiting its aggregation in the brain. It reduced oligomeric and fibrillar (plaque) amyloid, diminished hippocampal atrophy, improved cholinergic transmission, and stabilized cognition in preclinical and clinical studies. In this narrative review, current information on the efficacy and safety of tramiprosate, both in AD and in other neurocognitive disorders, is presented. Possible directions for future studies with tramiprosate are also discussed.

## Introduction

Alzheimer's disease (AD) has become the leading cause of cognitive impairment in an increasingly aging society (1). The slow but progressive course of cognitive deterioration, together with the lack of treatments that may halt the disease entails important functional dependence, particularly in the advanced stages of dementia. According to the World Health Organization (WHO) and Alzheimer Disease International (ADI) estimations, a 3-fold increase in the prevalence of dementia is expected by 2050, with 132 million people affected all over the world (2), which will produce enormous burden for healthcare systems and society (3, 4). Clearly, treatments are urgently needed, in order to stop, or at least delay, the course of cognitive deterioration and functional dependence in AD and other aging-associated neurocognitive disorders.

The aim of this paper is to review current information on the efficacy and safety of tramiprosate, in AD and in other neurocognitive disorders. In addition, possible directions for future studies with tramiprosate will be commented.

## Pathogenesis of Alzheimer's Disease

### Amyloid Pathology

AD is defined by progressive cognitive impairment and functional dependence, along with frequent behavioral abnormalities. Amyloid deposition is the earliest manifestation of the disease, giving a unique chemical and pathological signature to AD (5). The oligomerization of the amyloid peptide is postulated to drive the hyperphosphorylation of microtubule associated (tau) protein via tau misfolding, aggregation, and transynaptic transmission. While amyloid deposition develops very slowly, tau pathology is necessary for the occurrence of synaptic dysfunction and neuronal degeneration. In addition, a pivotal role of infectious agents and glial reaction has been suggested in the pathogenesis of AD (6, 7).

According to the amyloid hypothesis, selective enzymatic cleavage of the Aβ precursor protein (APP) generates Aβ peptide, oligomers, and fibrils, which form amyloid plaques and are eventually accumulated in the brain (5, 8). This accumulation results from an excessive production or a reduced clearance of Aβ, leading to a pathologic imbalance of the protein (9), being the 42-amino acid amyloid peptide (Aβ42) the main isoform found. Conformational transition from random-coil to β-pleated sheet shows greater tendency to aggregate and allows excessive deposition that eventually leads to neurotoxicity (10).

Since Aβ is crucial in AD pathogenesis, anti-amyloid treatment strategies are currently being investigated (11).

### Tau Pathology

Amyloid deposition in AD is commonly associated to an abnormal tau phosphorylation or aggregation. Tau is a protein that helps in maintaining the neuronal cell microarchitecture, promoting microtubule assembly and stabilization. In patients with AD, the hyperphosphorylation of tau increases its activity and promotes its accumulation, leading to neurofibrillary tangles (NFT) formation. In turn, NFT cause synapses loss, axonal transport impairment, and mitochondrial and cytoskeletal dysfunction (12). Additionally, tau misfolding and propagation through the synapses would also be crucial in the development of AD (6).

### Inflammation

In the last years, several authors have suggested a pivotal role of the immune system in AD pathogenesis; however, the relationship between immune response and amyloid accumulation is not completely elucidated. Innate immune response is localized at amyloid deposits and at NFT formation sites, promoting formation and aggregation of Aβ and NFT. Amyloid deposits are surrounded by astrocytes and microglial cells, involving complement cascade and microglia activation, release of pro-inflammatory mediators, and neuronal damage.

It is hypothesized that in AD, sustained immune response activation triggers a loop of continued signals that exacerbates amyloid deposit and tau pathology in a rapid and irreversible way (13, 14).

### Apolipoprotein E

Apolipoprotein E (ApoE) genotype is the major genetic risk factor for late-onset AD. The *APOE* ε4 allele confers a 4- to 12-fold higher risk of AD and lowers the age of onset by ~10–15 years (15, 16). The ApoE ε4 allele may also play a role in early-onset AD, delaying the onset of symptoms (17), and in other neurodegenerative diseases, where *APOE* ε4 status is a risk factor for co-pathology and poor evolution (18). ApoE protein binds to soluble Aβ and affects aggregation, transport, and clearance of amyloid within the central nervous system (CNS), increasing Aβ oligomers in the brain, which would contribute to the loss of dendritic spines thus accelerating memory impairment and leading to earlier cognitive decline in AD (16, 19, 20). Besides, preclinical studies showed that the expression of ApoE4 is associated with activation of a pro-inflammatory pathway in pericytes and blood-brain barrier breakdown, leading to neuronal uptake of neurotoxic proteins, as well as reductions in the blood flow (21).

In addition to *APOE*, over 20 risk loci have been identified in genome-wide association studies of late-onset AD or dementia, which are related to immunity, lipid metabolism, tau binding proteins, and amyloid precursor protein metabolism, showing the genetic and pathophysiological complexity of the disease and also highlighting the importance of comorbid or pleiotropic associations, gender differences, maternal history, and epigenetic factors (22).

### AD Biomarkers

AD prodromal biomarkers are crucial to establish early diagnosis and therefore early treatment. The diagnosis criteria of the National Institute on Aging and the Alzheimer's Association (NIA-AA) 2011 proposed Aβ42 and tau determination in CSF for AD diagnosis (23). Reduced cerebrospinal fluid (CSF) Aβ42 levels indicate brain deposition, which can be determined by CSF Aβ42/Aβ40 ratio or by amyloid positron emission tomography (PET) (24). Tau accumulation is associated with tangle formation and can be determined through Aβ/tau ratio in CSF and also by PET imaging (6, 25). Additional imaging biomarkers of downstream neuronal degeneration or injury include reduced 18-fluorodeoxyglucose (FDG) uptake in temporo-parietal cortex determined by PET and atrophy in the temporal lobe and medial parietal cortex determined by magnetic resonance imaging (MRI) (23).

Moreover, it is important to correlate biomarkers with the risk of dementia derived from AD, in particular, correlating biomarkers with low, intermediate, or high risk patients. In a cross-sectional study, Eliassen et al. researched the biomarker profile in patients with subjective cognitive decline (SCD), amnestic (aMCI), and non-amnestic (naMCI) mild cognitive impairment (MCI) patients. They observed elevated total-tau in aMCI and SCD groups. In addition, cortical glucose metabolism was found to be lower in aMCI, and naMCI patients showed a tendency for lower glucose metabolism. Finally, in aMCI and SCD groups was recorded a thinner entorhinal cortex (ERC). The authors concluded that naMCI and SCD patients showed a comparable biomarker profile, while aMCI showed the most pathologic biomarker burden (47.2% of patients with ≥2 biomarkers). Thus, aMCI showed more frequently AD pathological biomarkers representing a risk group (26).

Although exists an armamentarium of biomarkers to identify AD, additional biomarkers are still needed. A tiered approach has been proposed to screen candidate patients for biomarker assessment so that patients who do not show signs of AD pathophysiology may be excluded from cost biomarker performance. Clearly, the implementation of blood amyloid biomarkers in clinical practice would facilitate AD early detection, diagnosis, and follow-up in the future (27). Recently, neurofilament light chain (NfL) has been described as a sensible serum marker for the presymptomatic stages of AD (28).

## Tramiprosate

Tramiprosate is an orally administered small aminosulfonate compound that binds to Lys16, Lys28, and Asp23 of Aβ42. This binding results in the stabilization of Aβ42 monomers, thus reducing oligomeric and fibrillar (plaque) amyloid aggregation. The inhibition of oligomer formation and elongation provides neuroprotection against Aβ-induced subsequent deposition (10, 29–31).

Mechanisms of action of tramiprosate include effects on amyloid, but also anti-inflammatory effects as demonstrated in MCI patients (32, 33). A third mechanism of action is linked to the GABA-A receptor (GABA-AR). The molecular structure of tramiprosate is related to the neurotransmitter γ-amino butyric acid (GABA) structure, and it acts as a functional agonist (34, 35). The clinical effect of tramiprosate in cognitive and functional areas was mainly observed in AD patients which were homozygous for the ε4 allele of *APOE* (15, 16).

Although tramiprosate is a safe and usually well-tolerated drug, it is not yet authorized as a new AD drug. Besides, new tramiprosate prodrugs and metabolites are also being developed. ALZ-801 is an oral-administered tramiprosate prodrug with significantly improved pharmacokinetic variability and gastrointestinal tolerance. The phase I program reported good safety and tolerability results in healthy volunteers (36). On the other hand, 3-sulfopropanoic acid (3-SPA), the main metabolite of tramiprosate and ALZ-801, is an endogenous molecule present in the brain of patients with AD and other neurodegenerative disorders which has demonstrated anti-Aβ aggregation activity *in vitro*, with an efficacy comparable to tramiprosate. Thus, clinical improvements observed with tramiprosate or ALZ-801 may be partially due to the potential protective role of 3-SPA in the brain (36, 37).

## Literature Search Criteria

For this review, preclinical and clinical studies on tramiprosate were searched. For the selection of studies with relevant information on homotaurine, the following databases were consulted: Medline, Dimensions.ai, Google Scholar, and Cochrane Library, with the search completion date 01/05/2019. Search strings were performed on the above databases constructed from the term homotaurine and/or tramiprosate and all their equivalents or synonyms as input terms into the MeSH (Medical Subject Headings) database, the NLM controlled vocabulary thesaurus. These terms were conjugated with other terms derived from their pharmacological mechanism of action: “GABA Agonists,” “GABA receptor agonist,” or “glycosaminoglycan mimetic.” For screening, a restriction was made to those papers with IMRAD structure, published since 2006 (years of the first publication of tramiprosate phase II studies) and preferably in English. In the selection of studies, priority was given to prospective studies and reviews with adequate methodological quality. In addition, a secondary manual search of the bibliography of the studies finally selected was carried out to detect possible omissions that could be of interest for this work.

## Preclinical Studies on Tramiprosate

In preclinical studies, tramiprosate reduced oligomer formation and fibrillar (plaque) amyloid deposition in mouse model of AD. Treatment with tramiprosate induced a decrease of soluble amyloid protein levels and its deposition in the brain (plaque). Also, plasma Aβ levels declined in a dose-dependent manner indicating a role of tramiprosate in brain metabolism of Aβ or in its transport (10).

One preclinical study observed that tramiprosate (3-APS) favored tau polymerization in fibrillar aggregates, but these tau aggregates were not toxic in neuronal cultures. 3-APS also did not affect the binding of tau to microtubules and it promoted the decrease of tau-actin complexes that could be toxic for the cells (38).

In addition, GABA-dependent activity of tramiprosate has been reported. In rat primary neurons, tramiprosate binds with high affinity to the GABA-A receptors inducing caspase 3/7 activation. Accordingly, this effect is significantly decreased when neurons are pretreated with GABA-AR antagonists (35). In primary neurons treated with Aβ42, tramiprosate reduced caspase 3/7 and caspase 9 activities, both basal and Aβ42-induced. Also, in organotypic hippocampal slice cultures (OHCs) it decreased Aβ42-induced cellular mortality. Thus, in primary neurons, tramiprosate entails neuroprotective mechanisms against activation of caspases due to Aβ, and cellular mortality in OHCs; however, its effect on DNA damage is independent of the activation of GABA-AR (34).

Aβ induces long-term potentiation (LTP) inhibition in rat brain, and tramiprosate has shown no reverse this effect (39). Besides, it also inhibits Aβ42-induced extracellular signal-regulated protein kinases 1 and 2 (ERK1/2) activation by a GABA-A-independent mechanism (40). Overall, preclinical studies of tramiprosate demonstrate neuroprotective mechanisms involving both GABAergic and non-GABAergic pathways. A summary of preclinical studies on tramiprosate is showed in Table 1.

**Table 1:** Preclinical studies on trampirosate.

**Study**	**Model**	**Treatments**	**Outcomes**
Gervais et al. (10)	TgCRND8 mice*	30 or 100 mg/kg tramiprosate daily (s.c.) during 8–9 weeks.Controls were treated with sterile water.	Significant reduction in amyloid plaque in the brain. Significant decrease in the cerebral levels of soluble and insoluble Aβ40 and Aβ42. Dose-dependent reduction of plasma Aβ.
Galarneau et al. (35)	Rat primary neurons (*in vitro*)	Tramiprosate.3-H muscimol (control).	Tramiprosate bound to the GABA-AR with high affinity. Tramiprosate and 3-H muscimol induced a dose-dependent membrane depolarization and calcium flux. Tramiprosate and 3-H muscimol decreased basal and Aβ42-induced caspase 3/7 activity.
Azzi et al. (34)	Primary neurons and OHC (*in vitro*)	Tramiprosate.Muscimol (control).	Tramiprosate decreased basal and Aβ42-induced caspase 3/7 and caspase 9 activities. Decreased Aβ42-induced cellular mortality in OHC.
Krzywkowski et al. (39)	Rat hippocampal neurons (*in vitro*)	Tramiprosate	Tramiprosate prevented Aβ-induced inhibition of LTP.
Greenberg et al. (40)	Rat primary cortical/hippocampal neurons (*in vitro*)	TramiprosateMuscimol (control)	Tramiprosate inhibited Aβ42-induced ERK1/2 activation by a GABA-A-independent mechanism.
Santa-Maria et al. (38)	HEK293 tau cells (*in vitro*) SH-SY5Y cells (*in vitro*)	Tramiprosate, 3-APS, Alzhemed™	3-APS promoted abnormal aggregation of tau 3-APS did not affect the binding of tau to microtubules

*3-APS, 3-amino-1-propane sulfonic acid; ERK1/2, extracellular signal-regulated protein kinases 1 and 2; GABA-AR, γ-amino butyric acid A receptor; HEK, human embryonic kidney cells; LTP, long term potentiation; OHC, organotypic hippocampal slice cultures; s.c., subcutaneous; SH-SY5Y, human neuroblastoma cell line*.

*Alzheimer's disease model. Transgenic mice overexpressing mutant human APP at levels approximately 5-fold higher than endogenous murine APP.

## Clinical Studies with Tramiprosate in AD and other Neurocognitive Disorders

Results from a phase II trial demonstrate that tramiprosate safely reduces Aβ42 levels in the CSF of patients with mild-to-moderate AD. This CSF Aβ42 levels reduction together with clinical evaluations at long term indicate a role of tramiprosate in disease-modification. In addition, 3-months administration of tramiprosate was safe and well-tolerated (41). In the subsequent phase III study (Alphase study), tramiprosate did not show significant differences, but results were confounded by unexplained variance. In fact, a trend was observed for a treatment effect on the Alzheimer Disease Assessment Scale-cognitive subscale (ADAS-cog) (42). Moreover, pooled analysis of the two phase III trials (*n* = 2,025 patients with mild to moderate AD) considering ApoE4 allele distribution showed significant differences in ADAS-cog scores and a positive tendency on Clinical Dementia Rating Scale-Sum of Boxes (CDR-SB) in homozygote patients on 150 mg bid. ApoE4 heterozygotes showed an intermediate level of efficacy and non-ApoE4 patients did not show clinical benefits (29). Finally, subsequent re-analyses revealed most efficacy in the homozygote patients which were at the mildest clinical stage of disease (Mini-Mental State Examination 22–26). In those patients, tramiprosate showed benefits on ADAS-cog, CDR-SB, and DAD (Disability Assessment for Dementia) compared to placebo. Cognitive stabilization was observed over 78 weeks in the ADAS-cog, while both cognitive (ADAS-cog) and functional (DAD) effects increased over time (43).

The effect of tramiprosate on hippocampal volume was evaluated in a subgroup of patients (*n* = 312) from the Alphase study. In the final model analyses was demonstrated a significant link between tramiprosate dose and the reduction in hippocampus volume change (44, 45). Overall, the results of the phase III trials suggest a disease modifying effect of tramiprosate in AD, particular for the ApoE4/4 patients, at the earliest clinical stages of disease (46).

In patients with aMCI, which is very often an early clinical manifestation of AD, the effects of tramiprosate have been evaluated. Patients who met the criteria for aMCI (47, 48) and had a Clinical Dementia Rating (CDR) score of 0.5 (49) on tramiprosate showed less hippocampus and temporal lobe volume loss, which entails an improvement in short-term memory. Thus, tramiprosate supplementation protects against hippocampus atrophy and improves episodic short-term memory (33). In this line, a recent study evaluating tramiprosate administration in aMCI patients also showed improved short-term episodic memory performance in ApoE4/4 carriers. In addition to neuropsychological and functional assessments, cytokine levels were performed at baseline and after 1 year, and a significant decrease in IL-18 serum levels was observed, suggesting a drug-related anti-inflammatory effect (32). Another study in patients with symptomatic MCI showed an even earlier response. In patients with MCI according to Petersen criteria (48), 1-year administration of tramiprosate showed significant improvements from baseline expressed as MMSE (Mini Mental State Examination) score at months 8 and 12 in patients with aMCI, and at month 4 in those with naMCI (50). Finally, one study highlighted that tramiprosate could also modulate mechanisms of synaptic plasticity in aMCI patients, again diagnosed according to Petersen criteria (48). Treatment with 100 mg tramiprosate during 4 weeks showed measurable changes of short latency afferent inhibition (SLAI) suggesting a function in enhancing acetylcholine transmission, through the modulation of inhibitory cortical activity (51).

Tramiprosate efficacy has also been evaluated in Parkinson's disease (PD). One single-blind randomized controlled study that evaluated the safety and efficacy of tramiprosate in patients with PD and cognitive impairment showed that patients on treatment improved in the non-motor symptoms of the Unified Parkinson's Disease Rating Scale and presented a beneficial effect on excessive sleepiness measured by the Epworth Sleepiness Scale after 6 months, compared to controls (52).

In subjects with lobar intracerebral hemorrhage, a phase II double-blinded trial illustrated the pharmacokinetics and demonstrated the safety and tolerability of three different doses of tramiprosate. Nevertheless, testing of neurological function (measured by NIHSS), daily functioning (Barthel Index), cognition (ADAS-cog), and executive function (EXIT25) did not show significant changes among subjects during 12 weeks of treatment, compared to baseline (53). A summary of clinical studies with tramiprosate in AD and other neurocognitive disorders is showed in Table 2.

**Table 2:** Clinical studies on trampirosate.

**Study**	**Study design Number of patients**	**Disease**	**Treatment**	**Results**
Aisen et al. (41)	RCT *n* = 58	Mild-to-moderate AD	Placebo, 3-APS 50, 100, or 150 mg bid during 3 months	3-APS reduced CSF Aβ42 levels after 3 months of treatment
Alphase Study.Aisen et al. (42)	RCT *n* = 1,052	Mild-to-moderate AD	Placebo, tramiprosate 100 mg or tramiprosate 150 mg bid during 78 weeks	No significant treatment effect.Trend toward a treatment effect for ADAS-cog and less HV loss.
Abushakra et al. (29)	RCT *n* = 2,025 (pooled data from two phase III studies).	Three subgroups of patients with mild-to-moderate AD:ApoE4 homozygotes, ApoE4 heterozygotes and non-carriers	Placebo, tramiprosate 100 mg or tramiprosate 150 mg bid during 78 weeks	In ApoE4/4 homozygotes on 150 mg bid significant effects on ADAS-cog and positive trends on CDR-SB.Intermediate efficacy in ApoE4 heterozygotes and no benefit in non-carriers.
Abushakra et al. (43)	RCT *n* = 2,025 (pooled data from two phase III studies).	Three subgroups of patients with mild-to-moderate AD:ApoE4 homozygotes, ApoE4 heterozygotes and non-carriers	Placebo or 100 mg bid, or 150 mg tramiprosate bid	Highest efficacy (ADAS-cog, CDR-SB, and DAD scores) in ApoE4/4 homozygotes on 150 mg bid with mild disease.The mild subgroup showed cognitive stabilization with no decline over 78 weeks, both ADAS-cog and DAD effects increased over time.
Sabbagh et al. (46)	RCT A substudy of 257 subjects of Alphase Study.	Mild-to-moderate AD patients ApoE4 homozygotes	Placebo or 100 mg bid, or 150 mg tramiprosate bid	Statistically significant efficacy in the mild subgroup (ADAS and CDR scores).Some benefit at week 78 as indicated by DAD scores.
Spalletta et al. (33)	Controled study *n* = 33	aMCI	Tramiprosate 50 mg qd for 2 weeks and bid for the next year vs. untreated patients	Decreased volume loss in the left and right hippocampal tail, left and right fusiform gyrus, and right inferior temporal cortex.Improved short-term episodic memory performance.
Bossù et al. (32)	Open trial *n* = 20	aMCI	Tramiprosate 50 mg qd for 2 weeks and bid for the next year	Compared to baseline, patients with ApoE4 allele showed a significant decrease in IL-18 and improved short-term episodic memory performance.
Martorana et al. (50)	Observational retrospective study *n* = 245	aMCI and naMCI	Tramiprosate 100 mg qd	Significant improvements in cognitive decline (MMSE score) in patients with aMCI (at months 8 and 12), and naMCI (at month 4)
Martorana et al. (51)	Open trial *n* = 10	aMCI	100 mg tramiprosate during 4 weeks	Measurable changes of SLAI in aMCI patients. Potential role in enhancing cholinergic transmission by modulating the inhibitory cortical activity.
Gauthier et al. (44)	RCT Subset of 312 Alphase Study subjects.	Mild-to-moderate AD patients	Placebo, tramiprosate 100 mg or tramiprosate 150 mg bid during 78 weeks	Hippocampal atrophy slowing, beneficial effect on cognition (ADAS-cog)
Saumier et al. (45)	RCT *n* = 1,052 (Alphase study data)	Mild-to-moderate AD patients	Placebo, tramiprosate 100 mg or tramiprosate 150 mg bid during 18 months	ADAS-cog change scores and HV change correlate with vMRI.
Ricciardi et al. (52)	RCT *n* = 47	Parkinson disease	Tramiprosate 100 mg bid vs. untreated patients	Benefits on non-motor symptoms (UPDRS-I) and beneficial effect on excessive sleepiness (Epworth Sleepiness Scale).
Greenberg et al. (53)	RCT *n* = 24	Lobar intracerebral hemorrhage, with possible or probableCAA	Tramiprosate 50, 100, or 150 mg bid during 12 weeks	No significant changes on neurological function (NIHSS), daily functioning (Barthel Index), cognition (ADAS-cog), and executive function (EXIT25).

*3-APS, 3-amino-1-propanesulfonic acid; AD, Alzheimer's disease; ADAS, Alzheimer's Disease Assessment Scale; ADAS-cog, Alzheimer Disease Assessment Scale-cognitive subscale; aMCI, amnestic MCI; ApoE4, Apolipoprotein E4; ε4 allele of the apolipoprotein E gene; bid, twice daily; CAA, cerebral amyloid angiopathy; CDR-SB, Clinical Dementia Rating Scale, sum of boxes; DAD, Disability Assessment for Dementia; HV, hippocampus volume; MCI, Mild Cognitive Impairment; MMSE, Mini Mental State Examination; naMCI, non-amnestic MCI; NIHSS, NIH Stroke Scale; qd, daily; RCT, randomized controlled trial; SLAI, short latency afferent inhibition; UPDRS, Unified Parkinson's Disease Rating Scale; vMRI, volumetric magnetic resonance imaging*.

Safety of tramiprosate was excellent in all the clinical studies, with acceptable tolerability. In fact, mortality rate in the Alphase study was higher in the placebo group (4.0%) compared to the 100 mg bid (2.8%) and 150 mg bid (2.3%) groups. Patients with at least one adverse event for the placebo, 100 mg bid, and 150 mg bid groups were 92.1, 95.2, and 94.8%, respectively. Gastrointestinal disorders were the most common adverse events that led to discontinuation (3.6% of the patients). Nausea, vomiting, syncope, and weight loss seem to be dose-dependent; however, the incidence of those adverse events in the 100 mg bid group did not show significant differences compared to placebo. The ApoE4 patients displayed similar safety and tolerability profile compared to the complete sample and no amyloid-related imaging abnormalities (ARIAs) were observed (41, 43).

## Limitations of Tramiprosate Studies

Previous studies demonstrated that alteration of tau correlates better than amyloid deposition with the cognitive and behavioral manifestations of Alzheimer's patients (54, 55). Hence, it could be argued that, since tramiprosate mainly prevents amyloid aggregation, a clinical effect should not be expected. However, the efficacy of tramiprosate should be considered as multimodal, through different mechanisms, the reduction of amyloid deposition, the improvement of cholinergic transmission, an anti-inflammatory effect, and a probable neuroprotective effect, not favoring an abnormal aggregation of tau. Moreover, there is agreement regarding the convenience of early action on amyloid deposition and other mechanisms of AD to prevent tau pathology and cognitive deterioration (6, 56).

Tramiprosate studies have some limitations that could interfere in the clinical outcomes and study conclusions, mainly suboptimal study design (outcome measurements, lack or inadequate biomarkers used) and small sample size. Most importantly, time point for treatment inception, related to disease development, could also interfere in the study outcomes. Available data from failed phase III studies suggest that drug therapy should be started early, since patients with mild to moderate AD could be in advanced and irreversible stages of neurodegeneration at that point, being too late to improve their outcomes (41–43).

## Future Studies with Tramiprosate

Although tramiprosate is safe and well-tolerated, large controlled trials are still needed, particularly studies focusing on MCI and AD. Given the clinical heterogeneity of AD, the trials should be conducted in patients which are well-defined for the amyloid presence, tau protein, and neurodegeneration biomarkers (57). The heterogeneity of AD and MCI (clinical aspects, diagnosis, correlation with neuropathology, co-pathology in late onset AD, etc.) should be considered in future trials. Clearly, tramiprosate effects in neurodegeneration, inflammation, and hippocampus atrophy could be useful in patients at the early stages of AD to delay the onset of dementia (15).

Additionally, a major challenge will be to identify subgroups of highly respondent patients as well as the clinical and biological predictors of response. Nowadays, the main challenges for the management of neurocognitive disorders are to develop disease-modifying treatments and to improve symptomatic treatments. Tramiprosate has showed promising results in this line, and future directions with this drug are discussed below.

### Tramiprosate as a Disease-Modifying Agent

The pooled results of both phase III studies demonstrated the efficacy of tramiprosate in the treatment of ApoE4/4 carriers with mild AD through Aβ binding. These patients showed a stabilization of cognitive and functional performance, supporting the potential of this drug as a disease modifier (30, 43). Thus, future phase IV studies enriched with ApoE4/4 carriers focusing on aMCI and mild-moderate AD patients would be of interest.

A recent study established the ALZ-801 dose that provides bioequivalent exposures to 150 mg bid tramiprosate (ALZ-801 265 mg bid), which demonstrated cognitive and functional improvements in ApoE4/4 homozygotes. These data reinforced the development of phase III studies with ALZ-801 in ApoE4/4 carriers with AD or MCI (43, 58, 59).

Lewy body disease (LBD), Parkinson's disease dementia (PDD), vascular dementia (VaD) and frontotemporal dementia (FTD) patients are less frequently ApoE4 carriers than AD patients (60); however, these diseases are frequently associated with AD (61, 62). Hence, the subgroup of non-AD, but ApoE4/4 carriers, dementia patients would be candidates to receive tramiprosate or ALZ-801 treatment (60, 63–67).

Since clinical improvements observed with tramiprosate or ALZ-801 may be partially due to 3-SPA, it would be interesting to elucidate its role in ApoE4/4 carriers in future studies (36, 37). A summary of future investigations with tramiprosate, ALZ-801 and 3-SPA as disease modifiers on different neurocognitive disorders is given in Table 3.

**Table 3:** Future lines of research with tramiprosate, ALZ-801, and 3-APS.

**Disease-modifying treatments (amyloid pathology)**	
Tramiprosate	•Prospective observational studies with tramiprosate in aMCI and mild-moderate AD patients (phase IV)
ALZ-801	• aMCI and MA-MCI ApoE4/4 carriers (phase III, IV) • Mild AD ApoE4/4 carriers (phase III, IV) • ApoE4/4 carriers with non-AD neurodegenerative disorders: ∘ Lewy Body Disease (phase II, III, IV) ∘ Vascular dementia (phase II, III, IV) ∘ Frontotemporal dementia (phase II, III, IV) ∘ Parkinson-dementia (phase II, III, IV)
3-APS	• aMCI and MA-MCI ApoE4/4 carriers (phase II, III) • Mild and Moderate AD ApoE4/4 carriers (phase II, III)
**Symptomatic treatment (central cholinergic dysfunction)**	
ALZ-801	• aMCI (ALZ-801 vs. placebo) • Mild and moderate AD (ALZ-801 vs. placebo with CEI) • Parkinson disease subtype: early gait disturbances, visual hallucination, dysphagia, REM Sleep Behavior Disorder, olfactory impairment, cognitive deterioration (ALZ-801 vs. placebo, with or without CEI) • Huntington disease (ALZ-801 vs. placebo) • Brain injuries (ALZ-801 vs. placebo with or without CEI) • Vascular cognitive deterioration (ALZ-801 vs. placebo) • Vascular dementia (ALZ−801 vs. placebo, with or without CEI) • Hippocampal atrophy no-AD (PART, LATE) (ALZ-801 vs. placebo)

*3-APS, 3-amino-1-propanesulfonic acid; AD, Alzheimer disease; aMCI, amnestic MCI; ApoE4, Apolipoprotein E4, ε4 allele of the apolipoprotein E gene; CEI, cholinesterase inhibitor; LATE, Limbic-predominant age-related TDP-43 encephalopathy; MCI, MA-MCI, multi amnestic MCI; Mild Cognitive Impairment; PART, Primary age-related tauopathy*.

### Tramiprosate as a Symptomatic Treatment (Central Cholinergic Dysfunction)

Frequently, cognitive, behavioral, and motor disabilities are related to cholinergic dysfunction (68). Tramiprosate is a central GABA partial receptor agonist that modulates inhibitory cortical activity and improves cortical cholinergic transmissions in AD patients (51). In this line, tramiprosate or ALZ-801 could be useful for aMCI patients and mild/moderate AD patients to manage disease symptoms. Besides, other neurological entities with acetylcholine deficit could also benefit from treatment with tramiprosate.

Central cholinergic dysfunction has been reported in Parkinson's disease (PD) patients with early gait disturbances (69), visual hallucinations (70), REM sleep behavior disorder (71), dysphagia (72), cognitive decline (73), and olfactory impairment (74, 75). Thus, treatment with tramiprosate (or ALZ-801) alone or associated with cholinesterase inhibitors (CEI) may be considered in these PD subtypes to improve cholinergic transmission.

In VaD patients, deficits in the acetylcholine transmission or in the cortical and subcortical function are commonly present. Multiple factors influence production of vascular lesions in the basal forebrain cholinergic neuronal system (arterial hypertension, sustained hypoperfusion, cerebral small vessel disease, inflammatory reactions, oxidative stress, cerebral amyloid angiopathy damage, and ischemic cerebrovascular disease, among others). In these patients treatment with CEI may be useful (76), so exploring the potential benefit of tramiprosate (or ALZ-801), alone o combined, could also be interesting (66).

Traumatic brain injury (TBI) reduces cholinergic neurotransmission, decreases evoked release of acetylcholine, and alters cholinergic receptor levels. Treatment with CEI (galantamine) can reduce TBI pathology and improve cognitive function. Hence, TBI may be another candidate that could benefit from tramiprosate treatment (77).

In addition, it would be helpful to explore tramiprosate benefits in other neurodegenerative disorders which present dysfunction in cholinergic neurons and cholinergic transmission in the brain, such as Huntington's disease (78). The proposed future investigations with tramiprosate, ALZ-801, and 3-SPA on central cholinergic dysfunction are shown in Table 3.

Other non-AD neurodegenerative disorders with loss of episodic memory and selective hippocampal atrophy could also benefit from tramiprosate treatment. Thus, future studies with tramiprosate should be directed toward entities such as primary age-related tauopathy (PART) (79) and limbic-predominant age-related TDP-43 encephalopathy (LATE) (80).

Finally, since treatment combinations with different targets and pathophysiological mechanisms have been successful in other complex disorders such as HIV, this might also orientate future treatment in AD (81). Thus, it would be helpful to analyze the effects of combination therapies in the early stages, for example, treatment with tramiprosate and monoclonal anti-amyloid antibodies or CEI.

## Conclusions

Timely management of cognitive deterioration is of outmost importance in our aging society. Tramiprosate has been shown safe and effective in several neurocognitive disorders, particularly AD and MCI, where the results are consistent with a disease-modifying effect. Due to the heterogeneity of AD and MCI, definition of subgroups of responders should be crucial to implement future treatments. Clearly, large controlled trials, including well-characterized patients in terms of biomarkers and risk factors, are needed to confirm and extend the promising results of tramiprosate and 3-APS in the treatment of AD and other neurocognitive disorders.

## Author Contributions

All authors listed have made a substantial, direct and intellectual contribution to the work, and approved it for publication.

## Conflict of Interest

The authors declare that the research was conducted in the absence of any commercial or financial relationships that could be construed as a potential conflict of interest. The Editor declares he has received honoraria from Neuraxpharm, and declares no further competing interests that would impact the handling of this manuscript.
